# Role of matrix Metalloproteinases in pituitary adenoma invasion

**DOI:** 10.1186/s41016-017-0109-0

**Published:** 2018-02-09

**Authors:** Chengxian Yang, Xinjie Bao, Renzhi Wang

**Affiliations:** Department of Neurosurgery, Peking Union Medical College Hospital, Chinese Academy of Medical Sciences & Peking Union Medical College, Beijing, 100730 China

**Keywords:** Pituitary adenoma, Matrix metalloproteinase, Tumor invasion, Molecular biology

## Abstract

Though pituitary adenomas are benign tumors in most cases, a considerable fraction of PAs behave in a malignant-like manner and invade to the adjacent structures in sellar region, especially the cavernous sinuses. Cancer-cell invasion and metastasis remain a great challenge for physicians and surgeons in spite of emerging advances in drug therapy and surgical Treatmen*t. matrix* metalloproteinases, as a family of zinc-dependent endopeptidases, have long been known to be associated with tumor invasion and metastasis mainly via breaking down basement membrane in different tissues. Aberrant expression and activation of matrix metalloproteinases have been detected in invasive pituitary adenomas as in malignancy and correlated to tumor invasion. Therefore, matrix metalloproteinases are considered as promising biomarkers for predicting tumor behavior and even drug targets for novel therapeutic strategies. In this review, we give an overview of the expression, function, regulation and clinical prospects of matrix metalloproteinases, especially focusing on the biological network in which matrix metalloproteinases may be abnormally activated in promoting pituitary adenoma invasion.

## Background

Pituitary adenomas (PAs) are the second most common intracranial neoplasms with the prevalence of around 80/100,000, representing up to 25% of brain tumors [1–3]. Though PAs are commonly benign tumors, a considerable fraction of PAs, with the prevalence of 43% radiographically and 18% intraoperatively, behave in a malignant-like manner and invade to the adjacent structures including cavernous sinuses (CSs) and even internal carotid arteries [4, 5]. Though the magnetic resonance imaging-based Knosp grading system shows acceptable reliability and prognostic value [6–8], intraoperative visualization remains the gold standard for the diagnosis of invasive PA. Thus, preoperative detection of invasive PAs is of great significance for making appropriate surgical strategies.

PA invasion is the most common cause of incomplete surgical resection demanding further imaging follow-up, radiotherapy and even chemotherapy. Intraoperative findings of invasion to adjacent structures remain the gold standard in diagnosing PA invasion, whereas classification systems based on radiographic results are important predicting tools prior to surgery. In the present review, studies adopting intraoperative and radiographic standards are comprehensively reviewed. PA invasion stays a great challenge for most neurosurgeons though rich-experienced pituitary centers with the assistance of multiple surgical techniques can achieve gross total resection with a rate of up to 63.5% [9]. More tools are needed to assist the detection of PA invasion at an early stage. Taken together, there are challenges in early detection and effective therapy of PA invasion, whereas the mechanism remains poorly understood and requires further exploration. Accordingly, various biological molecules have been screened in order to discover a biological marker of relatively high sensitivity and specificity in predicting PA invasion.

Matrix metalloproteinases (MMPs), as zinc-dependent endopeptidases, consist of 24 members in mammals and mainly function by degrading the structural tissue components in extracellular matrix (ECM) [10]. MMPs are found at high levels of protein and transcription in various types of tumor. Previous researches have demonstrated that MMPs are associated with tumor invasion, metastasis and angiogenesis [11]. Aberrant expression and activation of MMPs are detected in invasive PAs, suggesting the potential role of MMPs in promoting PA invasion via its proteolytic activity [12, 13]. Emerging evidence suggests that MMPs are implicated in the infiltrative growth of PAs. In this review, we will summarize expression, function, regulation and clinical prospects of MMPs for a better understanding of PA invasion.

## Molecular basis of PA invasion

The medial wall (MW) of CS, which is located in the lateral side of pituitary gland, is penetrated by invasive PAs. The mainstream view is that MW of CS mainly consists of two distinct layers including pituitary capsule (PC) and dura mater (DM). The existence of histologic defect in MW of CS enabling PA invasion has been denied anatomic researches. Instead, the interaction between MW components and biological properties of PAs are believed to account for invasion. Peker et al. [14] was the first to investigate the collagen expression pattern of ECM in sellar region. They found that PC and DM share collagen I and II expressions in common while collagen III, IV and V are differentially located in PC alone. Knappe et al. [15] revealed a relatively different expression pattern of collagen in perisellar ECM but also certified the fact that collagen IV is densely expressed in PC. In contrast, Kawamoto et al. [13] demonstrated that collagen IV is the main functional component of DM in PAs, and therefore proposed that type IV collagenases are involved in PA invasion. Different from previous studies, Ceylan et al. [16] defined MW and PC as two distinct membrane structures and found high concentrations of collagen IV in both membranes. Though results of these studies concerning anatomic structures and collagen expressions of MW are slightly controversial, these findings all point out that collagen IV is the key component of ECM in MW of CS which may be activated by type IV collagenase, such as MMP-9 and MMP-2, in PA invasion.

## MMPs function in PA invasion

### Proteolysis

The destruction of integrity of ECM by proteolysis is considered as the main underlying mechanism of invasion in tumor behavior. Proteolytic activation of MMPs from a latent to an active form is regulated in a biochemical procedure termed as cysteine-switch in which a cysteine residue in the pro-domain compounding to zinc-binding region is removed to unmask the proteolytic site [17, 18]. Kawamoto et al. [12, 13] were the first to put forward the thesis that collagen IV is the main functional component of DM in PAs, and further discovered that immunohistochemical staining with MMP-9, a type IV collagenase, is strongly positive in invasive PAs but negative in noninvasive ones, associating MMP-9 and CS invasion. This series of great groundbreaking value first introduced the concept of MMP-induced invasion to PA invasion researches. In addition, MMP-2, known as another gelatinase of MMP family except for MMP-9, is also associated with tumor invasion in PAs [19, 20].

### Angiogenesis

Angiogenesis is a key biologic factor involved in tumor proliferation, growth, invasiveness, and other cellular processes. MMPs play a vital role in angiogenesis and PA invasion [21]. Jugenburg et al. [22] performed a quantitative morphologic study to disclose the vascular supply of PAs by measuring percentages of capillary area, number of vessels per field, percentage of endothelial cells and numbers of endothelial cells per field. They found that the vascular density is lower in PAs than in normal pituitary tissues, and is almost the same between invasive and noninvasive PAs. In contrast, Turner et al. [23] detected higher vascular density in invasive macroprolactinoma compared with noninvasive macroprolactinoma, suggesting the potential role of microvessels in PA invasion. The relationship between angiogenesis and PA invasion still remains conflicting.

However, MMP-9 overexpression and angiogenesis are positively correlated in invasive PAs though the mechanism remains unclear. Turner et al. [24] explored the role of MMP-9 in regulating tumor behavior of PAs, and they found MMP-9 overexpression in invasive macroprolactinomas compared with noninvasive ones. Based on their previous studies, they further figured out the positive correlation between MMP-9 and angiogenesis in tumor invasiveness [23, 24]. Pan et al. [25] also revealed higher vascular density in invasive PAs than in noninvasive ones and established the positive correlation between MMP-9 and angiogenesis.

Furthermore, increased expression of MMP-14 is observed at mRNA and protein levels in invasive PAs [26, 27]. Hui et al. [27] tried to explain the underlying mechanism by which MMP-14 contributes to PA invasion via silencing MMP-14 gene in ATT20 cell lines. They demonstrated that the downregulation of MMP-14 is accompanied by suppressed expressions of PTTG, VEGF and TGF-β which participate in angiogenesis in both physiological and pathological conditions. They concluded that MMP-14 expression is likely to be upregulated by increased PTTG expression, resulting in higher VEGF expression and subsequent angiogenesis-mediated PA invasion [27].

## Dysregulation of MMPs in invasive PAs

### Protein kinase

Protein kinase C (PKC) is a family of protein kinase enzymes playing important roles in cellular signal transduction. PKC system-dependent MMP-2 and MMP-9 upregulations are associated with invasion and metastasis in different kinds of tumors. Aberrant expressions of MMP-2 and MMP-9 induced by PKC-related signaling pathways are found to promote invasion in glioma, breast cancer, melanoma, gastric cancer, colon cancer, hepatocellular cancer and et al. [28–34]. Previous studies have found significantly higher activity and expression of PKC in PAs than normal pituitary tissues as well as distinctive overexpression of PKC in invasive PAs compared with noninvasive ones [35, 36]. Furthermore, a single point mutation of PKC-α termed as D294G is screened out and confirmed in PAs with more invasive phenotypes, indicating the pivotal role of PKC in PA invasion [37, 38]. In nonfunctioning PAs and HP75 cell lines, Hussaini et al. [39] observed higher expression and activity of MMP-9 and detailed the role of PKC, especially its isoenzymes (PKC-α and PKC-δ) in elevating MMP-9 expression and activity. Addition of phorbol-12-myristate-13-acetate (PMA) can activate PKC and result in increased MMP-9 expression. PKC inhibitors and gene silencing can block PA invasion induced by PMA-mediated MMP-9 overexpression. Hence, they proposed the combination of MMP-9 and PKC inhibitors as novel strategies for treating invasive PAs [39].

### Receptor tyrosine kinase

The discoidin domain receptors (DDRs) are unique receptor tyrosine kinases characterized by binding collagens as ligands [40]. Cell-collagen interaction is activated by DDRs in regulating cancer cell behavior. DDRs consist of two distinct subtypes, DDR1 and DDR2. Yoshida et al. [41] investigated the expression and function of DDR1 in HP-75 cell lines by clone transfection and gene silencing. They revealed that PA invasion is enhanced by the binding of DDR1-collagen I which elevates the cellular secretion of MMP-2 and MMP-9. A further research demonstrated that hypoxia increases the expression of DDR1 at mRNA and protein levels [42]. It is also confirmed that hypoxia-associated overexpression of DDR1 enhances the secretion of MMP-2 and MMP-9 and promotes PA invasion [42].

### Cytokine

Emerging evidence has demonstrated the promoting role of inflammatory interleukin 17 (IL-17) and IL-17 receptors (IL-17R) in cancer invasion and metastasis, suggesting IL-17/IL-17R as promising targets for cancer immunotherapy [43]. In contrast, the study concerning IL-17/IL-17R axis in PAs is limited due to the benign tumor behavior. Qiu et al. [44] performed a research detecting relatively higher expression of IL-17, IL-17R and MMP-9 at mRNA and protein levels and meanwhile found the positive correlation of MMP-9 with IL-17 and IL-17R, respectively. Serum IL-17 concentration is significantly higher in invasive PAs than in noninvasive ones, suggesting IL-17 as a biomarker for invasiveness prediction in PAs. These findings warrant a deeper exploration into the relation between IL-17/IL-17R axis and MMPs in invasive PAs. Previous immunohistochemical results showed weak expression of IL-6 in PAs and normal tissues and demonstrated no significant correlation between IL-6 and MMP-2 or MMP-9 [45]. However, integrative proteomics and transcriptomic data with bioinformatics analysis detected IL-6 as an activated upstream regulator in invasion of pituitary null cell adenomas [46]. Moreover, the data suggested that IL-6/JAK2/STAT3 pathway contributes to tumor invasion by elevating MMP-9 expressions [46].

### Tumor suppressor gene

Hepatocellular carcinoma, downregulated 1 (HEPN-1) gene is a novel tumor suppressor gene first described in human hepatocellular carcinoma (HCC). Moh et al. [47] revealed that silenced HEPN-1 gene is frequent in HCC and that transfection of HEPN-1 gene into HepG2 cell lines exerts antineoplastic effect. In HCC, miRNA-21 can suppress HEPN-1 expression resulting in carcinogenesis [48]. In somatotroph PAs, HEPN-1 silencing is associated with aggressive tumor behavior and is found to promote invasiveness via upregulation of MMP-2 and MMP-9 in GH3 and GT1.1 cells [49]. Contradictorily, miRNA −21 is downregulated in corticotropinomas, suggesting its different biologic role in PAs compared with HCC [50].

### Wnt signaling pathways

Previous studies have revealed that Wnt signaling pathways are critical for the development of pituitary gland and tumorigenesis of PAs [51]. Wnt/β-catenin signaling pathways are termed as canonical signaling participating in transcription changes. Wnt-4 overexpression is observed in most PAs, and Wnt-4 excessive activation is inversely correlated to tumor invasion [52]. In β-catenin knockdown PA cells, reduced invasiveness and a drastic reduction of MMP-2/9 are detected simultaneously, suggesting that MMP-2/9-medicated PA invasion may be in the downstream of Wnt/β-catenin signaling pathway [53].

### Tissue inhibitor of MMPs

Tissue inhibitors of MMPs (TIMPs) are endogenous regulators of MMP function via binding specific sites of MMPs and therefore considered as potential inhibitors of PA invasion. TIMPs consist of 4 members in human, which are TIMP-1, TIMP-2, TIMP-3 and TIMP-4. Beaulieu et al. observed an inverse correlation between TIMP-2 and TIMP-3 levels and tumor invasiveness. Sun et al. [54] revealed that expression of TIMP3 in mRNA and protein levels was negatively correlated with tumor invasiveness in Cushing disease. In prolactinomas, TIMP-2 was found to be a marker for tumor invasion and recurrence [55]. However, the mechanism of TIMPs and interaction between TIMPs and MMPs in PA invasion have not been clarified.

**Fig. 1:**
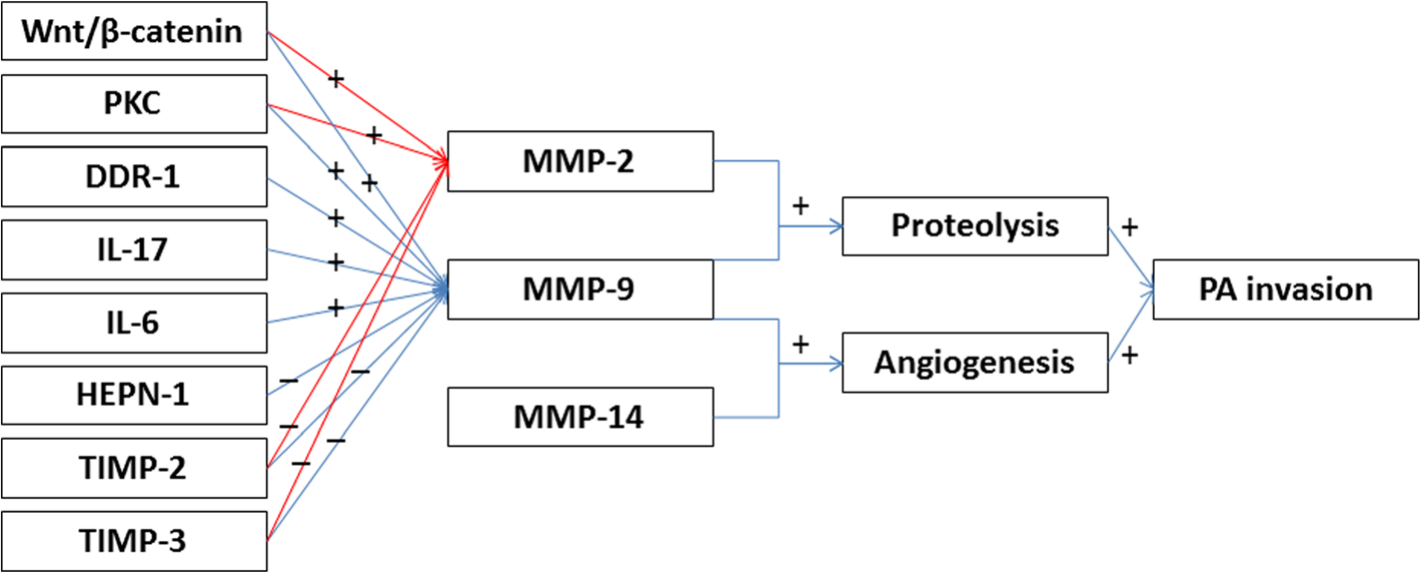
Schematic overview of function and regulation of MMP family members in PA invasion

Taken together, MMPs regulation network has not been clarified (Fig. 1). We think that there may be following reasons: (i) There have been cushing’s disease dogs and prolactinoma rats used for imaging, surgery and drug studies [56–58]. However, animal models of different PA subtypes have not been stably established [58, 59], and alternative PA cell lines are easily affected by experimental conditions. (ii) tissue samples of human PAs are difficulty to collect in most research centers, and therefore molecular studies with large sample size are quite limited. (iii) Underlying mechanism of MMPs in promoting PA invasion may be not specific enough in nature leading to indefinable correlation between aberrant molecular expression and invasive behavior.

## Potential role of MMPs in diagnosing invasive PAs

In light of the positive association between MMPs and PA invasion, MMPs are recommended as promising biomarkers for detecting invasive PAs [20, 55, 60–62]. Among all the candidate biomarkers of MMPs, MMP-2 and MMP-9 are the most studied ones. Gong et al. [60] strongly recommended MMP-9 as a reliable biomarker for detecting invasive PAs and evaluating the extent of invasiveness. In their test, significant increased MMP-9 expression and activity are seen and certified in invasive PAs in contrast to noninvasive ones. Moreover, MMP-9 can distinguish the extent of invasiveness regardless of tumor types, size and status (primary or recurrent), paving the way of MMP-9 to clinical practice. Moreover, MMP-9 expression may be affected by dopamine agonist drugs in prolactinomas, indicating that MMP-9 can reflect the response to drugs [55]. Liu et al. [19] demonstrated that high MMP-2 mRNA and protein levels correlate with PA invasiveness without respect to tumor size and hormone secretion. They proposed MMP-2 as a powerful tool for distinguishing the invasive potential of PAs. These findings demonstrate the potential roles of MMP-9 and MMP-2 as biomarkers. However, there is no related study giving out the cut-off point, specificity and sensitivity of serum MMP-9 or MMP-2 concentrations in predicting PA invasion.

## Conclusion

MMPs are considered as promising biomarkers and future drug targets contributing to early detection and improved prognosis of invasive PAs. The clinical value of MMPs in predicting PAs invasion is of great significance and worthy conducting a perspective study to correlate the blood levels of MMPs and infiltrating tendency and extent of PAs. Taken together, further studies, in terms of both molecular biology and clinical evidence, are warranted to elucidate the role of MMPs in invasive PAs.

## Abbreviations

CS: Cavernous sinus
DDR: Discoidin domain receptor
DM: Dura mater
ECM: Extracellular matrix
HCC: Human hepatocellular carcinoma
HEPN-1: Hepatocellular carcinoma, downregulated 1
IL: Interleukin
MMP: Matrix metalloproteinase
MW: Medial wall
PA: Pituitary adenoma
PC: Pituitary capsule
PKC: Protein kinase C
PMA: Phorbol-12-myristate-13-acetate
TIMP: Tissue inhibitors of MMP
